# Fluoroquinolone Efficacy against Tuberculosis Is Driven by Penetration into Lesions and Activity against Resident Bacterial Populations

**DOI:** 10.1128/AAC.02516-18

**Published:** 2019-04-25

**Authors:** Jansy Sarathy, Landry Blanc, Nadine Alvarez-Cabrera, Paul O’Brien, Isabela Dias-Freedman, Marizel Mina, Matthew Zimmerman, Firat Kaya, Hsin-Pin Ho Liang, Brendan Prideaux, Jillian Dietzold, Padmini Salgame, Radojka M. Savic, Jennifer Linderman, Denise Kirschner, Elsje Pienaar, Véronique Dartois

**Affiliations:** a Public Health Research Institute, New Jersey Medical School, Rutgers, The State University of New Jersey, Newark, New Jersey, USA; b Department of Medicine, New Jersey Medical School, Rutgers, The State University of New Jersey, Newark, New Jersey, USA; c Department of Bioengineering and Therapeutic Sciences, University of California San Francisco, San Francisco, California, USA; d Department of Chemical Engineering, University of Michigan, Ann Arbor, Michigan, USA; e Department of Microbiology and Immunology, University of Michigan Medical School, Ann Arbor, Michigan, USA

**Keywords:** MDR-TB, fluoroquinolone, lesion-centric pharmacology, moxifloxacin, tuberculosis

## Abstract

Fluoroquinolones represent the pillar of multidrug-resistant tuberculosis (MDR-TB) treatment, with moxifloxacin, levofloxacin, or gatifloxacin being prescribed to MDR-TB patients. Recently, several clinical trials of “universal” drug regimens, aiming to treat drug-susceptible and drug-resistant TB, have included a fluoroquinolone. In the absence of clinical data comparing their side-by-side efficacies in controlled MDR-TB trials, a pharmacological rationale is needed to guide the selection of the most efficacious fluoroquinolone. The present studies were designed to test the hypothesis that fluoroquinolone concentrations (pharmacokinetics) and activity (pharmacodynamics) at the site of infection are better predictors of efficacy than the plasma concentrations and potency measured in standard growth inhibition assays and are better suited to determinations of whether one of the fluoroquinolones outperforms the others in rabbits with active TB. We first measured the penetration of these fluoroquinolones in lung lesion compartments, and their potency against bacterial populations that reside in each compartment, to compute lesion-centric pharmacokinetic-pharmacodynamic (PK/PD) parameters. PK modeling methods were used to quantify drug penetration from plasma to tissues at human-equivalent doses. On the basis of these metrics, moxifloxacin emerged with a clear advantage, whereas plasma-based PK/PD favored levofloxacin (the ranges of the plasma AUC/MIC ratio [i.e., the area under the concentration-time curve over 24 h in the steady state divided by the MIC] are 46 to 86 for moxifloxacin and 74 to 258 for levofloxacin). A comparative efficacy trial in the rabbit model of active TB demonstrated the superiority of moxifloxacin in reducing bacterial burden at the lesion level and in sterilizing cellular and necrotic lesions. Collectively, these results show that PK/PD data obtained at the site of infection represent an adequate predictor of drug efficacy against TB and constitute the baseline required to explore synergies, antagonism, and drug-drug interactions in fluoroquinolone-containing regimens.

## Abstract (precis)

Fluoroquinolones represent the pillar of multidrug-resistant tuberculosis (MDR-TB) treatment, with moxifloxacin, levofloxacin, or gatifloxacin being prescribed to MDR-TB patients. Recently, several clinical trials of “universal” drug regimens, aiming to treat drug-susceptible and drug-resistant TB, have included a fluoroquinolone.

## TEXT

Newer-generation fluoroquinolones (FQs) are pivotal drugs in the treatment of multidrug-resistant tuberculosis (MDR-TB) (1, 2) and have been included in “universal” regimens under clinical evaluation (3–5). While moxifloxacin (MXF) and gatifloxacin (GTX) produced disappointing results in first-line treatment-shortening trials (6–8), treatment success is associated with the use of fluoroquinolones in MDR-TB patients (1) and fluoroquinolone resistance is in turn associated with poor clinical outcome (9, 10). The successful inclusion of a fluoroquinolone in upcoming trials may thus depend on selecting the most promising drug within the class and on understanding its efficacy in different lesion types.

Fluoroquinolones are broad-spectrum antibiotics that inhibit DNA supercoiling and disrupt DNA replication by trapping gyrase in Mycobacterium tuberculosis (and topoisomerase IV in other bacteria) on DNA as ternary complexes that block the movement of replication forks (11). Gyrase is an ATP-dependent enzyme that acts by creating transient double-stranded DNA breaks, and fluoroquinolones exert their lethal effect by freezing the gyrase-damaged DNA complex. MXF, GTX, and levofloxacin (LVX) are among the most potent FDA-approved later-generation fluoroquinolones, with activity both against the enzyme and in whole cells (12), providing a rationale for their use in TB patients by WHO guidelines (13). Ofloxacin is not considered here since it is a racemic mixture consisting of 50% LVX and 50% of its inactive enantiomer dextrofloxacin.

Little knowledge is available to guide the choice of a fluoroquinolone by clinicians, and therefore the nomination of a “best-in-class” choice to treat TB—the fluoroquinolone that would lead to durable cure in the highest proportion of MDR-TB patients—remains a matter of debate (14). An in-depth study of fluoroquinolone pharmacodynamics (PD) in experimental TB chemotherapy would help define the role of fluoroquinolones in the treatment of human TB. In addition, their broad spectrum of activity allows the comparison of pharmacokinetic-pharmacodynamic (PK/PD) parameters predictive of efficacy between Mycobacterium tuberculosis and other pathogenic species (15).

In early bactericidal activity (EBA) trials where the three drugs were compared side by side, 14 days of MXF (400 mg), high-dose LVX (1,000 mg), or GTX (400 mg) daily treatment achieved potent and similar effects on bacterial burden in sputum (16). High-dose LVX has a cost advantage and possibly a more favorable toxicity profile than the other two (17), although in-depth clinical validation regarding long-term use is required to verify this trend (14). In mice, the pharmacokinetic/pharmacodynamic (PK/PD) parameter that drives efficacy of the fluoroquinolones is the area under the concentration-time curve (AUC) over 24 h in the steady state divided by the MIC (AUC/MIC ratio) (18). Applying these metrics in patients, high-dose LVX exhibits a slight PK/PD advantage (19). However, two recent studies suggested a small but potentially relevant advantage for MXF as follows: (i) in a standard mouse model of TB infection, MXF at the human-equivalent dose of 400 mg showed efficacy superior to that of high-dose LVX (20), and (ii) using a systems pharmacology approach combining experimental and computational methods, Pienaar et al. generated a model that simulates granuloma formation and function, fluoroquinolone plasma pharmacokinetics, and spatial and temporal tissue distributions and integrates extensive *in vitro* and *in vivo* data. Comparing the three drugs side by side on identical simulated granulomas, they concluded that MXF may have a clinical advantage over LVX (21). In two independent studies of experimental chemotherapy in mice with TB by the same authors, MXF appeared more potent than LVX (22, 23). Retrospective analyses and small prospective trials have been conducted to differentiate selected fluoroquinolones based on treatment outcome but have failed to identify a winner (24–27). Such studies are particularly challenging owing to the complexity and diversity of MDR-TB background regimens.

Drug distribution and efficacy studies in animal models of TB disease have shown that reaching adequate drug concentrations at the sites of infection is critical in achieving sterilization and clinical utility (28–31). Learning from these lessons, we hypothesized that PK/PD analyses performed at the site of disease may help differentiate the fluoroquinolones. Here, we leveraged the rabbit model of active TB to compare the lesion-centric PK/PD data and efficacies of MXF, LVX, and GTX in the absence of a confounding background regimen. Rabbits infected with Beijing strain HN878 develop a spectrum of pulmonary lesions similar to those observed in human TB, and the trajectories of these lesions diverge within the same animal as disease progresses, similarly to results seen with nonhuman primates (32, 33). In this model, lesion-based PK/PD metrics clearly differentiated the three drugs and predicted a substantial advantage for MXF while the three fluoroquinolones were found to be equivalent by measure of plasma PK relative to traditional potency values. We then confirmed these findings in side-by-side efficacy studies at human-equivalent doses, providing an experimental basis to guide the selection of a fluoroquinolone for MDR-TB patients and in future clinical trials.

## RESULTS

### Comparative pharmacokinetics-pharmacodynamics of MXF, LVX, and GTX.

The efficacy of fluoroquinolones is concentration dependent and driven by the area under the concentration-time curve relative to the MIC (AUC/MIC) (18, 34), one of the three conventional PK/PD parameters (35). To generate comparable PK/PD parameters for the three antibiotics, we measured the MIC and minimum bactericidal concentrations (MBCs) of MXF, LVX, and GTX against M. tuberculosis side by side. Mean exposure (AUC_[0–24]_) ranges at clinically approved doses were derived from a published study where the three fluoroquinolones were profiled in parallel in TB patients (19). By measure of plasma PK/PD, i.e., unbound drug exposure (*f*AUC) in plasma relative to MIC or MBC, the three fluoroquinolones cannot be clearly differentiated, with LVX potentially emerging ahead of the other two by a small margin (Table 1). Thus, based on clinical plasma PK/PD metrics, LVX may be expected to deliver superior efficacy.

**TABLE 1:** Plasma PK/PD parameters in TB patients receiving the WHO-recommended doses of MXF, LVX, or GTX

Drug	Dose (mg/day)	MIC_90_ (mg/liter)^*a*^	MBC (mg/liter)^*b*^	AUC_[0–24]_ (mg*h/liter)^*a*^ median (SD)	*f*AUC_[0–24]_ range (mg*h/liter)^*c*^	*f*AUC/MIC range^*c*^	*f*AUC/MBC range (mg*h/liter)^*c*^
Moxifloxacin	400	0.5	0.3	57 (12)	23–43	46–86	77–143
Levofloxacin	1,000	1.0	0.9	129 (106)	74–258	74–258	82–287
Gatifloxacin	400	0.5	0.5	40 (7)	22–39	44–78	44–78

a Data are from reference 19.

b Data are from reference 47.

c Data were computed using MIC_90_ values from reference 19, MBC values from reference 47, and average human plasma protein binding values obtained as part of this work (Table S2).

Since we have previously shown that lesion-based PK/PD parameters may predict the clinical efficacy of ethambutol better than plasma PK/PD (30), we next measured (i) the concentration of MXF, LVX, and GTX in lung lesions and (ii) the potency of each fluoroquinolone against bacterial populations found in cellular and necrotic granulomas and cavities to generate lesion-centric PK/PD indices.

**FIG 1:**
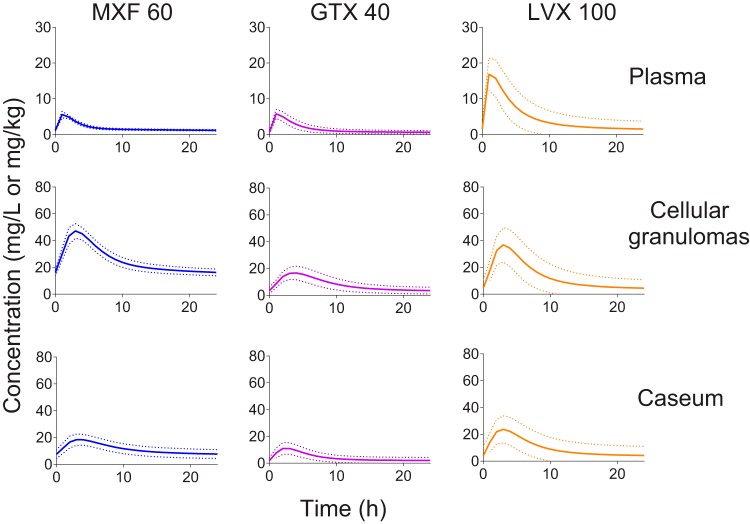
Modeling and simulation of the distribution of moxifloxacin (MXF), gatifloxacin (GFX), and levofloxacin (LVX) at human-equivalent doses in cellular granulomas and in caseum. Solid and dashed lines show means and standard deviations, respectively, for 100 lesions of each type. PK profiles in plasma and cellular granulomas were simulated using drug concentration data generated by high-pressure liquid chromatography and tandem mass spectrometry (LC/MS-MS) in plasma and tissue homogenates. PK profiles in caseum were simulated using data generated by laser capture microdissection coupled to LC-MS/MS.

With this aim, we used the rabbit model of active TB, which presents with pathological features similar to human disease and which recapitulates the drug penetration from plasma to lesions seen in TB patients (29, 33, 36). First, the concentration-time curves of MXF, LVX, and GTX were established in plasma at increasing doses (see Fig. S1A and B in the supplemental material), and the corresponding areas under the concentration-time curves (AUCs) were calculated in order to infer the human-equivalent dose to be used in subsequent efficacy studies. Clinical AUCs for MXF, GTX, and LVX were retrieved from references 19, and 37–39. Next, we measured the distributions of MXF, LVX, and GTX from plasma into uninvolved lung tissue, cellular and necrotic granulomas, and cavity caseum (see Data Set S1 in the supplemental material) in rabbits that were infected with M. tuberculosis HN878 until mature lesions developed, or 13 to 22 weeks postinfection. The data were analyzed and modeled to (i) generate PK parameters to identify the human-equivalent dose of each fluoroquinolone (see Table S1 and Fig. S1C in the supplemental material), (ii) establish coefficients of penetration from plasma into cellular and necrotic lesions and caseum (Table S1), and (iii) predict drug concentrations in cellular lesions and caseum at the steady state. The PK model was then used to simulate the concentration-time profile of each fluoroquinolone in plasma and in 100 cellular and necrotic granulomas at the steady state (Fig. 1), at doses that reproduce free plasma AUCs (*f*AUCs) observed in TB patients receiving WHO-recommended daily doses (MXF 400 mg, LVX 1,000 mg, and GTX 400 mg [19]). Differential protein binding in rabbit and human plasma (Table S2) was accounted for in the calculation of *f*AUCs. At the human-equivalent doses of 60 mg/kg of body weight for MXF, 100 mg/kg for LVX, and 40 mg/kg for GTX, MXF achieved higher concentrations and AUC levels in cellular and necrotic lesions than LVX and GTX (Fig. 1), whereas the plasma AUC of LVX was highest among the three drugs in patients and rabbits (Table S3).

**FIG 2:**
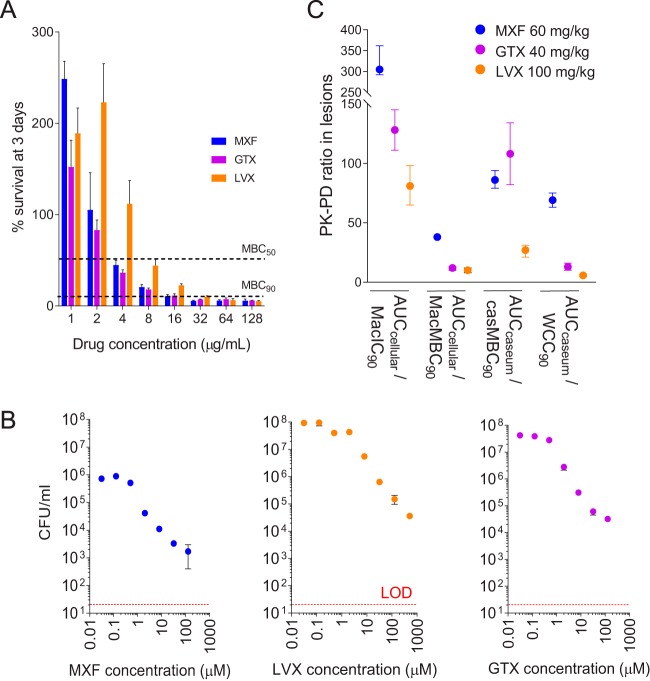
Lesion-centric pharmacokinetic-pharmacodynamic (PK/PD) parameters of the fluoroquinolones calculated using potency measured against intracellular M. tuberculosis in primary macrophages and extracellular M. tuberculosis in rabbit caseum. (A) Activated macrophages were infected with M. tuberculosis and treated with the fluoroquinolones for 3 days as indicated. Relative growth or survival rates on day 3 compared to pretreatment are shown (means ± standard deviation [SD], *n* = 3). (B) Bactericidal activity of the fluoroquinolones against *ex vivo* M. tuberculosis in cavity caseum, where tuberculous bacilli are rich in lipid inclusions and exhibit phenotypic drug resistance (45). Data are expressed as CFU per milliliter of homogenized caseum (diluted 1:3 in water), plotted on a log scale (means ± SD, *n* = 3). LOD, limit of detection. (C) Lesion-specific PK/PD parameters calculated as the ratios between the drugs’ area under the curve in cellular lesions (AUC_cellular_) or caseum (AUC_caseum_) and the drugs’ inhibitory or cidal activity against the corresponding intracellular and extracellular M. tuberculosis bacilli. MacIC_90_, concentration that inhibits 90% of growth in macrophages; MacMBC_90_, concentration that kills 90% of M. tuberculosis bacilli in macrophages (A); WCC_90_, Wayne cidal concentration or concentration that kills 90% of extracellular M. tuberculosis under anaerobic conditions (47); casMBC_90_, concentration that kills 90% of M. tuberculosis bacilli in *ex vivo* rabbit caseum (45) (B). Means ± SD of data from 100 simulated granulomas or caseous foci are shown.

We next asked how the concentrations achieved in cellular lesions and caseous foci of necrotic lesions compared to the concentrations required to either kill or inhibit growth of bacterial populations known to reside in relevant lesion compartments. In human and rabbit TB, extracellular M. tuberculosis bacilli are found in the caseous (necrotic) foci of granulomas and cavities, and intracellular bacilli are mostly present in macrophages and, to a lesser extent, in other immune cells that make up the cellular rim of lesions (32, 40–42). To account for the drug tolerance phenotype conferred by lesion microenvironments (43, 44), the potency of each fluoroquinolone was measured against intracellular M. tuberculosis bacilli replicating in bone marrow-derived macrophages (Fig. 2A) and extracellular M. tuberculosis present in *ex vivo* caseum retrieved from cavities of rabbits with active TB (Fig. 2B), where M. tuberculosis bacilli exhibit high intrabacterial lipid inclusion content and are profoundly drug tolerant (45). MBCs representing hypoxic conditions (46) were retrieved from the literature (47). These potency values, together with simulated fluoroquinolone AUCs in cellular lesions and caseum, were used to infer lesion-specific PK/PD parameters for MXF, LVX, and GTX (Table 2) (Fig. 2C). By these lesion-centric metrics, MXF clearly emerged as the fluoroquinolone achieving higher PK/PD targets in lesions.

**TABLE 2:** Lesion PK/PD parameters in rabbits receiving the human-equivalent dose of MXF, LVX, or GTX^*a*^

Drug	MacIC_90_ (mg/liter)	MacMBC_90_ (mg/liter)	WCC (mg/liter)	CasMBC_90_ (mg/liter)	Simulated AUC_cellular[0–24] _ mean (95% CI) (mg*h/liter)	Simulated AUC_caseum[0–24] _ mean (95% CI) (mg*h/liter)	h 2, h 6, h 12 caseum/cell ratio^*b*^
MXF	2	16	4	3.2	609 (584–635)	276 (251–301)	0.36, 0.79, 0.62
GTX	1.5	16	18	2.2	192 (166–218)	103 (83–123)	0.93, 0.98, 0.51
LVX	4	32	18	4.0	325 (259–392)	238 (181–295)	0.76, 1.16, 1.02

a MacIC_90_ and MacMBC_90_, MIC_90_ and MBC_90_, respectively, against intracellular M. tuberculosis in murine bone marrow-derived macrophages (this work); WCC, Wayne cidal concentration (the concentration which kills 90% of viable bacilli under hypoxia-induced nonreplicating conditions) (from reference 47); casMBC_90_, MIC_90_ (1-log kill) against *ex vivo* M. tuberculosis from cavity caseum; AUC_cellular[0–24]_, area under the concentration-time curve from 0 to 24 h in cellular granulomas at the human-equivalent dose as determined from the computational model; AUC_caseum[0–24]_, area under the concentration-time curve in caseum; CI, confidence interval.

b Data were determined by laser capture microdissection and HPLC coupled to tandem mass spectrometry (55, 67).

### Pharmacokinetic-pharmacodynamic target attainment in plasma, lung, and lesions at human-equivalent doses.

**FIG 3:**
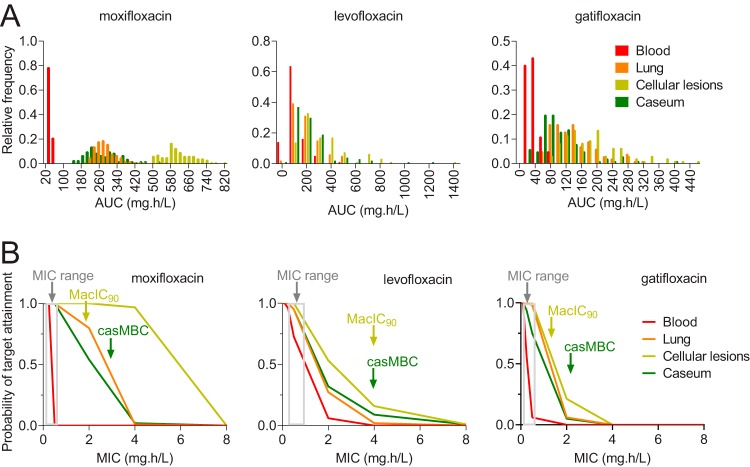
(A) Steady-state AUC_[0–24]_ distributions in plasma, uninvolved lung, cellular lesions, and caseum of rabbits receiving human-equivalent doses of MXF, LVX, and GTX as indicated. The PK variability was introduced by sampling 100 plasma PK parameter sets from the distributions given in Table S1. (B) Probability of target attainment for a PK/PD target AUC_[0–24]_/MIC of 125 to achieve 90% of maximal kill (15, 18, 19). The gray areas indicate the range of MIC as reported by Angeby et al. (48). Red, orange, olive, and green lines indicate the probability of target attainment in plasma, uninvolved lung, cellular lesions, and caseum, respectively. The minimum concentration required to achieve 90% growth inhibition of intracellular M. tuberculosis (MacIC_90_) and the concentration required to kill 90% of nonreplicating M. tuberculosis bacilli in caseum (casMBC) are indicated by arrows. These two potency values were determined using M. tuberculosis strains H37Rv and HN878; thus, the impact of potency variability on target attainment and efficacy predictions is unknown.

To compare the levels of efficacy and sterilizing potential of the fluoroquinolones in lung and lesion compartments, we performed a probability of target attainment (PTA) analysis using (i) AUC ranges and PK variability obtained during model building at human-equivalent doses in plasma, lung, cellular lesions, and caseum (Table S3); (ii) PD parameters derived from published MIC ranges and EUCAST epidemiologic cutoff values (ECOFF) (48); and (iii) the published total AUC/MIC target of 125 to achieve 90% of maximum killing effect (Emax) (18, 19, 34). PTA was higher in lung tissue than in plasma, was higher for MXF than for GTX and LVX, and was higher in uninvolved lung and cellular lesions than in caseum for the three fluoroquinolones (Fig. 3). In cellular lesions, MXF achieved 100% target attainment at potency values up to 4 μg/ml. This analysis confirmed the superior therapeutic potential of MXF and predicted higher sterilization activity in cellular than necrotic lesions for all three fluoroquinolones in the rabbit model.

### Comparative efficacies of MXF, LVX, and GTX in the rabbit model of active TB.

To determine whether fluoroquinolone efficacy is driven by lesion-centric PK/PD parameters, we leveraged the active-TB rabbit model to directly quantify the effect of drug treatment on the bacterial burden (quantified as CFU counts) present in individual lesions. Rabbits were infected for 8 weeks until mature lesions developed. The average bacterial burden prior to treatment initiation (8 weeks postinfection) was 1.1 × 10^7^ CFU per lung pair (interquartile range, 1.9 × 10^5^ to 1.5 × 10^7^ CFU per lung pair). From then on, rabbits were treated with vehicle only or with MXF, GTX, or LVX at the WHO-recommended human-equivalent doses (MXF 60 mg/kg, GTX 40 mg/kg, and LVX 100 mg/kg, determined by steady-state simulations) for 8 weeks, with groups of rabbits analyzed after 4 and 8 weeks. To ensure on-target fluoroquinolone exposure at the steady state, three to five rabbits per group were randomized to a pharmacokinetic (PK) therapeutic drug monitoring substudy after 2 to 3 weeks of treatment (Data Set S2). The average PK parameters of MXF, GTX, and LVX closely matched the simulation-predicted AUCs (Table S3) and fell well within the range of human exposure achieved in TB patients at the steady state (Table S4).

**FIG 4:**
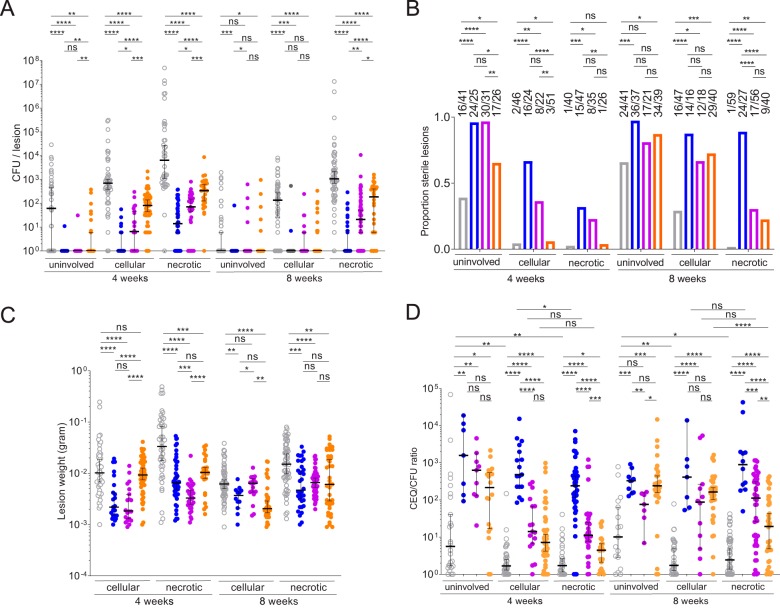
Comparative efficacies of the three fluoroquinolones in the rabbit model of active TB. (A) Effect of MXF (blue), GTX (purple), and LVX (orange) daily treatment at human-equivalent doses on bacterial burden in uninvolved lung, cellular lesions and necrotic lesions, compared to vehicle-treated controls (gray), at 4 and 8 weeks of treatment. Data are presented as median ± 95% confidence interval; *n* = 4 to 6 rabbits or 13 to 59 lesions per lesion category and treatment group, with data determined using the two-tailed Mann-Whitney U (nonparametric) test. *, *P* < 0.05; **, *P* < 0.01; ***, *P* < 0.001; ****, *P* < 0.0001; ns, not significant. (B) Effect of MXF (blue), GTX (purple), and LVX (orange) treatment on sterilization of lung tissue and lesions, shown as proportions of sterile uninvolved lung samples and lesions. Actual ratios are shown above each bar. Sample size and *P* value categories correspond to those described for panel A. Fisher's exact test (two-sided) was performed for comparisons of treated versus untreated group pairs. (C) Effect of fluoroquinolone treatment on lesion weight. Sample size and statistical analysis correspond to those described for panel A. (D) Effect of MXF (blue), GTX (purple), and LVX (orange) treatment on bacterial kill in lung and lesions, calculated as the ratio between CEQ (chromosome equivalents; cumulative burden) and CFU (actual live burden at the time of sampling). Data points with undetectable CEQ were removed from the analysis since the CEQ LOD is approximately 100 (33) and the CFU LOD is 3 to 5. *n* = 4 to 6 rabbits or 7 to 54 lesions per lesion category and treatment group (2/4 MXF-treated rabbits had few lesions left after 2 months of treatment, hence the reduced number of lesions in this group), with data determined using the two-tailed Mann-Whitney U (nonparametric) test. *, *P* < 0.05; **, *P* < 0.01; ***, *P* < 0.001; ****, *P* < 0.0001; ns, not significant.

The bacterial burden of individual lesions was quantified after 4 and 8 weeks of daily treatment (Data Set S3). All three fluoroquinolones significantly reduced bacterial burden in cellular and necrotic lesions after 1 and 2 months of treatment. We also measured the drug effect in seemingly uninvolved lung where cellular microlesions, not detectable macroscopically, were present. Interestingly, the uninvolved lung samples showed a high rate of self-sterilization, leading to nonsignificant drug effect after 2 months. In cellular lesions at 1 month and necrotic lesions at 1 and 2 months, MXF caused higher reductions in bacterial burden than GTX, which in turn was superior to LVX. After 2 months, the median bacterial burden in cellular lesions was below the detection limit in all three drug groups (Fig. 4A; see also Data Set S3). The ability of each fluoroquinolone to fully sterilize cellular and necrotic lesions followed the same trend (MXF > GTX > LVX), although the differences between the drugs were not always statistically significant (Fig. 4B). Higher proportions of sterile lesions were observed in the microlesion and cellular lesion categories than in necrotic lesions, where bacterial load is higher at treatment start (33). Cellular lesions underwent full sterilization both as a result of immune pressure alone (in vehicle-treated controls) and due to immune pressure and drug effect combined (Fig. 4B). Overall, the bacterial loads that remained in necrotic lesions were higher than those seen in cellular lesions at the completion of treatment, regardless of the fluoroquinolone used (Fig. 4A). Together, these results are consistent with the preferential partitioning of fluoroquinolones in cellular compared to caseous lesion compartments (Fig. 1). Within this 2-month time frame, we did not detect emergence of fluoroquinolone resistance in any of the lesions. We also measured the drug effect on lesion weight: each fluoroquinolone caused a decrease in average lesion weight, but only MXF consistently achieved statistically significant weight reduction of cellular and necrotic lesions after 1 and 2 months of treatment (Fig. 4C).

In previous efficacy studies where bacterial burden was measured in individual lesions, chromosome equivalents (CEQ), representing a surrogate of the cumulative bacterial burden of a given lesion, were also quantified in lesions, allowing the calculation of immune- and drug-mediated killing within lesions over time, reflected by the CEQ/CFU ratio (33, 49). To compare the kill rates achieved by each fluoroquinolone in cellular and necrotic lesions, we quantified CEQ in all lesions and uninvolved lung samples (see Fig. S2; see also Data Set S3). In drug-naive rabbits, we found minimal immune-mediated killing in cellular and necrotic lesions (with CEQ approximately equal to CFU) and significantly higher kill rates in uninvolved lung suspected to contain microlesions, although those rates were low at, on average, around 5-fold to 10-fold (Fig. 4D). In drug-treated animals, MXF consistently achieved higher kill rates than GTX and LVX in lung, cellular, and necrotic lesions after 1 and 2 months of treatment. The differences were not statistically significant in uninvolved lung at either time point or in cellular lesions at 2 months. Note that the results of analysis of the CEQ data set in this and another recent study by our group (33) indicated that M. tuberculosis DNA is not equally stable in lung and lesions exposed to different drugs or left untreated. Treatment with MXF and, to a lesser extent, with GTX resulted in loss of CEQ in uninvolved lung and cellular lesions over time at a level higher than that in necrotic lesions (Fig. S2). As bacteria are killed, macrophage and neutrophil bystanders likely phagocytose M. tuberculosis cell remnants and degrade free DNA. Accordingly, the level of this phenomenon appeared to be reduced in necrotic lesions where caseum is largely devoid of active immune cells. The levels of killing capacity of MXF and GTX were similar in cellular and necrotic lesions, while LVX was significantly less active in necrotic than in cellular lesions at the late time point (Fig. 4D; see also Fig. S2). Overall, these results were consistent with CFU and lesion sterilization data (Fig. 4A and B) and further confirmed the superiority of MXF.

### Suboptimal MXF underperforms in necrotic lesions.

**FIG 5:**
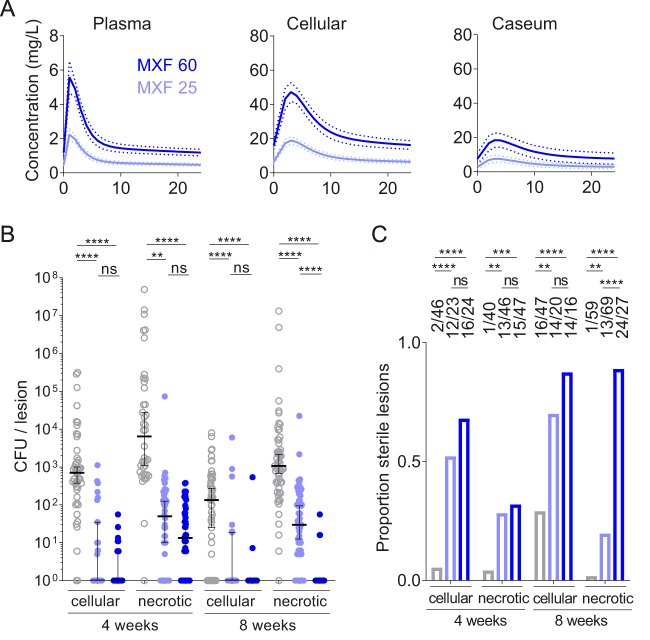
Impact of suboptimal MXF plasma pharmacokinetics on tissue distribution and bacterial burden in lung and lesions. (A) Simulated plasma and lesion PK profiles at the ideal human-equivalent dose of 60 mg/kg (dark blue) and the suboptimal dose of 25 mg/kg reproducing exposure in the lower 5th percentile of the AUC range (light blue). (B and C) Effect of canonical and suboptimal MXF exposure on bacterial burden in uninvolved lung, cell, and necrotic lesions (B) and lesion sterilization (C). Color codes are identical in panels A, B, and C. Data are presented as median ± 95% confidence interval; *n* = 4 (MXF-treated) to 6 (vehicle-treated) rabbits or 16 to 59 lesions per lesion category and treatment group as determined using the two-tailed Mann-Whitney U (nonparametric) test. *, *P* < 0.05; **, *P* < 0.01; ***, *P* < 0.001; ****, *P* < 0.0001; ns, not significant.

The clinical pharmacokinetics of the fluoroquinolones are prone to interindividual variability similar to that seen with most antibiotics (19, 37, 39). In addition, coadministration of rifampin (50) or rifapentine (51) causes a significant decrease in MXF AUC. We thus evaluated the impact of suboptimal MXF exposure on lesion PK parameters and bacterial burden reduction, using the same study design as that described above. A rabbit dose of 25 mg/kg was selected to reproduce human exposure at the lower end of the AUC spectrum (51). The lesion PK model was used to simulate MXF concentrations over time in plasma (Fig. S1C) and cellular lesions and caseum (Fig. 5A) and to calculate AUC values (Table S3). To assess MXF exposure at 25 mg/kg and the steady state, four rabbits were randomized to a therapeutic drug monitoring substudy after 3 weeks of treatment. The average AUC matched the simulation-predicted AUCs (Table S4) and fell within the lower 5th percentile of MXF AUCs reported in TB patients (51, 52). Suboptimal MXF exposure significantly reduced bacterial load (Fig. 5B) and increased sterilization of both cellular and necrotic lesions compared to untreated controls but, as expected, the magnitude of this effect was lower than in rabbits receiving the standard MXF dose of 60 mg/kg (Fig. 5B). However, the differences in bacterial burden and lesion sterilization between standard and suboptimal MXF doses were statistically significant only in necrotic lesions after 8 weeks of treatment, suggesting that lower-than-standard MXF concentrations can be tolerated in cellular lesions without significantly compromising efficacy. Sustained sterilization of necrotic lesions required ideal MXF exposure (Fig. 5C). No MXF-resistant colonies emerged on plates containing 0.5 mg/liter MXF, or 2 times the MIC of M. tuberculosis HN878.

## DISCUSSION

Fluoroquinolones are widely used in the treatment of MDR-TB and have recently been included in several universal regimen trials for the treatment of drug-susceptible TB and MDR-TB, but whether the efficacy of one drug is superior to that of another has been difficult to assess clinically in the context of multidrug regimens. This is in contrast with the wealth of PK/PD and research information pertaining to the treatment of infections caused by fast-growing organisms with fluoroquinolones (15). In the absence of evidence-based guidance for TB, LVX is often preferred to MXF due to MXF’s quantitative (QT) prolongation and potential for cardiotoxicity (53). On the other hand, long-term use of LVX has been associated with musculoskeletal disorders in pediatric populations (54). On the basis of their exposure in patient plasma and *in vitro* potency in standard MIC assays, MXF and GTX have similar efficacy potentials and LVX shows a wide and slightly higher range of *f*AUC/MIC (Table 1). Against a disease with complex pathology such as TB, we and others have hypothesized that lesion-based rather than plasma-based PK/PD parameters drive drug efficacy (30, 31). Here we have compared the lesion-centric PK/PD and efficacy of three fluoroquinolones in the rabbit model of active TB, which recapitulates the major immunopathology features of human TB lesions (33). We have found that plasma PK/PD parameters are associated with a low probability of target attainment (PTA) for the three fluoroquinolones, consistent with previous analyses (15, 18), but also that PTA was higher in cellular lesions where fluoroquinolones accumulate, reminiscent of what was observed for ethambutol (30). This was most striking for MXF, which exhibits the highest ratios between exposure in lesions and potency against the resident bacterial populations, or lesion PK/PD parameters. This in turn translated into efficacy superior to that of LVX and GTX in the rabbit model. Interestingly, a multiscale systems pharmacology approach to model lesion penetration and drug killing in *in silico* granulomas also revealed the superior potential of MXF (21). A recent comparative efficacy study in mice indicated that high-dose LVX is less effective than high-dose MXF against both fluoroquinolone-resistant and -susceptible M. tuberculosis strains in mice (20). Thus, *in silico*, *in vitro*, and *in vivo* findings each point toward MXF as the fluoroquinolone with the most attractive pharmacological profile when given as monotherapy.

When we simulated the low MXF exposure seen in patients with poor pharmacokinetics (51, 52), we found that suboptimal MXF concentrations were tolerated in cellular lesions without significant compromise of efficacy. This was consistent with a recent study in which we showed that fluoroquinolones preferentially partition within macrophage- and foamy macrophage-rich pockets inside the cellular rims of TB lesions (55), a niche that hosts a reservoir of M. tuberculosis bacilli (41). However, low MXF exposure resulted in significantly reduced bacterial clearance and lesion sterilization in necrotic lesions, in line with lower penetration of MXF into caseum and higher tolerance of the resident bacteria (45, 55).

Therapeutic drug monitoring of rabbits with TB disease on FQ treatment revealed moderate interanimal variability (see Data Set S2 in the supplemental material) which did not correlate with average bacterial burden, suggesting that interday and interlesion PK variability most likely overrides interanimal differences in absorption, metabolism, and elimination. We did not observe emergence of resistance over the 2-month treatment course, despite plasma *f*AUC/MIC values (range, 18.4 to 36.9; see Data Set S2) that fell well below the target value of 53 established in the hollow-fiber system (34) as well as below the AUIC (single area under the inhibitory concentration-time curve) value of 125 proposed for Gram-negative pathogens (56, 57). This suggested that longer exposure to sustained subtherapeutic drug levels is required for MXF resistance to emerge in M. tuberculosis *in vivo*. In addition, development of resistance may occur only in lesions with levels of bacterial burden higher than those found in rabbits that have been infected for 8 weeks (33).

Fewer lesions were collected in a subset of rabbits after 8 weeks of treatment, due to treatment-associated reduced pathology and lesion resolution (see Table S5 in the supplemental material; see also the data corresponding to the MXF arm at 8 weeks in Fig. 4D). This may have led to underestimation of the effect of the most efficacious drug(s)—and thus of the efficacy differential between MXF and the two other FQs—since lesions that are largely resolved are not macroscopically detectable and were missed in the analysis.

Both in this study and in a previous study by our group (33), careful analysis of CEQ data indicated that M. tuberculosis DNA is not equally stable in cellular and necrotic lesions, whether drug treated or not. In cellular lesions, as bacteria are killed, M. tuberculosis cell remnants and DNA are likely phagocytosed by activated macrophages and neutrophil bystanders. In necrotic lesions, where the bacterial load is mostly extracellular in caseum—devoid of active immune cells—DNA of dead bacteria is more stable. Loss of CEQ appears more prominent in cellular lesions and uninvolved lung treated with MXF than in those treated with GTX or LVX, which may be due to superior killing by MXF. One could hypothesize that MXF is more effective than other fluoroquinolones in causing DNA cleavage (58), leading to more frequent double-stranded breaks in the bacterial DNA and, in turn, making it more susceptible to further degradation. Interestingly, the range of CEQ/lesion ratios in LVX-treated rabbits was narrower than in drug-naive and MXF- or GTX-treated animals (see Fig. S2 in the supplemental material), and the median remained constant over time, suggesting that LVX treatment limits both M. tuberculosis DNA degradation and bacterial replication or that the two processes are in equilibrium. Overall, these observations suggest that bacterial growth and kill processes are less dynamic in LVX-treated lesions and that LVX may exert a more static effect than the two other fluoroquinolones.

Multiple retrospective analyses of clinical outcomes in MDR-TB patients have failed to detect the potential superiority of MXF, with various possible explanations. First, fluoroquinolones represent 1 of 4 to 7 drugs in the regimens of MDR-TB patients, and partner drugs are tailored to the susceptibility profile of each patient, making direct comparisons challenging and affecting the effect size that can be statistically detected (24–26). Second, the plasma levels of MXF (which undergoes phase II biotransformation) but not GTX or LVX (both of which are excreted unchanged) are reduced by coadministration of rifampin and rifapentine (51, 59). Thus, in the context of first-line-drug substitutions, MXF may be at a disadvantage. Finally, Drusano and colleagues have shown antagonism between MXF and rifampin in the hollow-fiber system (60) and in mice (61), and their results were recently corroborated by *in vitro* studies (62). The present results constitute the baseline required to explore synergies, antagonism, and drug-drug interactions in FQ-containing regimens.

MXF has been recently included in clinical trials that evaluate the efficacy of universal regimens against drug-susceptible and MDR-TB (5, 63) (ClinicalTrials.gov identifier NCT02193776). This reinforces the need to confirm the potential advantage of MXF over GTX and LVX at currently approved doses in controlled clinical trials. The contrasting performance of fluoroquinolones in first-line (6–8) versus MDR (1, 64) regimens suggests that the partner agents and their bactericidal properties may play a critical role in revealing or masking fluoroquinolone contribution.

## MATERIALS AND METHODS

### *In vitro* assays.

Human and rabbit plasma protein binding and rabbit caseum binding were measured using rapid equilibrium dialysis (RED) (Thermo Fisher Scientific) as described previously (65). The minimum bactericidal concentration assay against M. tuberculosis found in rabbit caseum (casMBC) was performed as described previously (45). Briefly, rabbit caseum was homogenized and incubated with MXF, GTX, and LVX at concentrations ranging from 0.03125 to 128 μM for 7 days and then plated on Middlebrook 7H11 agar for CFU enumeration, including no-drug controls. casMBC_90_ is defined as the minimum concentration that killed 90% of bacteria residing in caseum.

To measure fluoroquinolone activity against intracellular M. tuberculosis, bone marrow-derived macrophages from C57BL6 mice were infected with strain Erdman M. tuberculosis expressing a luciferase plasmid used as a measure of growth, at a multiplicity of infection of 10 to 1. After 4 h of infection, extracellular bacteria were washed off and the medium was supplemented with appropriate drugs as follows: cells were treated in triplicate with 2-fold increasing doses of MXF, LVX, and GTX from 1 μg/ml to 128 μg/ml. Luminescence readings were taken at days 0, 1, 2, and 3. Data were plotted at day 3 after drug treatment, and fluorescence readouts were correlated with bacterial growth. The MBC_90_ was defined as the minimum bacterial concentration that killed 90% of intracellular bacteria relative to day 0. Data are presented as percentages of day 0 luminescence after adjustment for well-to-well variation.

### Rabbit infection, pharmacokinetics, and chemotherapy.

All animal studies were performed in biosafety level 3 facilities and approved by the Institutional Animal Care and Use Committee of the New Jersey Medical School, Rutgers University, Newark, NJ. Female New Zealand White (NZW) rabbits (Millbrook Farm, Concord, MA), weighing 2.2 to 2.6 kg, were maintained under specific pathogen-free conditions and fed water and chow *ad libitum*. The rabbits were infected with M. tuberculosis HN878, using a nose-only aerosol exposure system as described previously (66).

### Plasma and lesion-centric pharmacokinetic studies.

Pharmacokinetic (PK) studies were conducted as described previously (30). Briefly, at defined time points from 16 to 20 weeks postinfection, rabbits received a single dose of 100 mg/kg MXF (Chemieliva Pharmaceuticals, China) or 75 mg/kg LVX or 100 mg/kg GTX (Chem-Impex Intl, IL), formulated in 40% sucrose and polyethylene glycol (PEG) 400 (90:10), by oral gavage. Drugs were extracted from tissue homogenates by adding an acetonitrile/methanol mixture containing specific internal standards (100 ng/ml MXF-d4, 200 ng/ml LVX-d8, and 10 ng/ml verapamil). Samples were vortexed and centrifuged, and the supernatants were stored for analysis by high-pressure liquid chromatography (HPLC) coupled to tandem mass spectrometry (LC/MS-MS).

### High-pressure liquid chromatography and mass spectrometry (LC/MS-MS) methods.

NZW rabbit control plasma treated with K_2_EDTA was obtained from Bioreclamation Inc. (Westbury, NY). Control (untreated) rabbit lungs, granulomas, and caseum were collected in-house. Standard curve and quality control spiking solutions were prepared by diluting 1 mg/ml dimethyl sulfoxide (DMSO) stocks of MXF, LVX, and GFX in acetonitrile/water (1:1). A 20-μl volume of neat spiking solutions was added to 20 μl of drug-free plasma or control lung tissue homogenate and then extracted by addition of 180 μl of acetonitrile/methanol (1:1) containing 100 ng/ml MXF-d4, 200 ng/ml LVX-d8, and 10 ng/ml verapamil. MXF-d4 and LVX-d8 were purchased from Toronto Research Chemicals. The mixtures were subjected to vortex mixing and centrifuged at 4,000 rpm for 5 min. The supernatant was recovered for LC/MS-MS analysis. LC/MS-MS analysis was performed on a Sciex Applied Biosystems 4000 triple-quadrupole mass spectrometer coupled to an Agilent 1260 high-pressure liquid chromatography (HPLC) system to quantify LVX, GTX, and MXF levels in the samples. Chromatography was performed with an Agilent Zorbax SB-C8 column (4.6 by 50 mm; particle size, 3.5 μm) using reverse-phase gradient elution. All gradients used 0.1% formic acid–Milli-Q deionized water for the aqueous mobile phase and 0.1% formic acid–acetonitrile for the organic mobile phase. Multiple-reaction monitoring of parent/daughter transitions in electrospray ionization (ESI)-positive mode was used to quantify the analytes. The compounds were ionized using ESI-positive mode and monitored using masses of MXF (402.2/358.1), MXF-d4 (406.2/362.1), LVX (362/318.5), LVX-d8 (370/326.6), GFX (376/261.2), and verapamil (455.4/165.2). Sample analysis was accepted if the concentrations of the quality control samples were within 20% of the nominal concentration. Data processing was performed using Analyst software (version 1.6.2; Applied Biosystems Sciex).

### Laser capture microdissection.

For laser capture microdissection, whole lesions (including the surrounding uninvolved lung) were collected and frozen as previously described (67). Tissue sections (12-μm thick) were cut from gamma-irradiated rabbit lung biopsy specimens using a Microm HN505 N instrument (Walldorf, Germany) and were thaw-mounted onto stainless steel slides (matrix-assisted laser desorption ionization–mass spectrometric imaging [MALDI MSI]) or standard glass microscope slides for hematoxylin-eosin (H&E) staining. Levofloxacin-d8 (TRC, Toronto, Ontario, Canada), gatifloxacin-d4 (TRC, Toronto, Ontario), or moxifloxacin-d4 (Clearsynth, Ontario, Canada) was added to the matrix at 5 pmol/μl as an internal standard for LVX, GTX, or MXF, respectively. Microdissected areas were immediately transferred to sealed containers and stored at −80°C prior to analysis by LC/MS-MS.

### Lesion-centric efficacy studies.

New Zealand White rabbits were infected by the aerosol route as described above. Starting 8 weeks postinoculation, groups of 8 to 9 rabbits received 60 mg/kg MXF or 40 mg/kg GTX or 100 mg/kg LVX or vehicle only 6 days a week (to give the animals a 1-day rest from daily sedation) for either 4 weeks (4 rabbits) or 8 weeks (4 to 5 rabbits). At each time point, rabbits were sedated with ketamine and xylazine, were euthanized by the use of pentobarbital (Euthasol), and underwent necropsy. From each rabbit, approximately 10 cellular and 10 necrotic lesions, as well as five pieces of uninvolved (not containing macroscopically visible lesions) lung tissue, were dissected, weighed, and processed as described previously (33). The smallest dissected lesions were approximately 1 mm in diameter. The surrounding uninvolved lung tissue, which can be distinguished by its reddish color, was carefully shaved off with a sterile scalpel. Fewer lesions were collected from a subset of rabbits after 8 weeks of treatment as a consequence of treatment-associated lesion resolution and reduced pathology. All tissue samples were homogenized in either 250 μl or 500 μl of phosphate-buffered saline (PBS), based on sample size. Serial dilutions of tissue homogenates were made in PBS supplemented with 0.025% Tween and plated on Middlebrook 7H11 agar. Undiluted homogenate was plated on Middlebrook 7H11 agar supplemented with 0.5 mg/liter MXF. Plates were incubated at 37°C for 4 to 6 weeks before determining the final CFU counts. The lower limit of detection was 5 CFU/lesion. After approximately 3 weeks of daily treatment, blood was collected from the central ear artery of three to five rabbits per treatment group predose and at 0.5, 1, 2, 4, 6, and 24 h following oral gavage to assess exposure in infected rabbits at the steady state and to compare the results with the average exposure achieved in TB patients receiving the WHO recommended dose.

### Computational modeling methods.

**(i) Model structure and implementation.** The full computational methodology was described previously (21) and briefly reviewed here. To predict and compare the levels of fluoroquinolone efficacy in granulomas, we developed the mechanistic computational model *GranSim* (68–70) (http://malthus.micro.med.umich.edu/GranSim/). *GranSim* is a 2-dimensional spatiotemporal hybrid model of granuloma formation and function that incorporates macrophage and T cell recruitment, migration, and interaction; secretion and diffusion of chemokines and cytokines; M. tuberculosis growth and phagocytosis; and caseation. The emergent behavior of *GranSim* simulations is the formation of spatiotemporal *in silico* granulomas. In the context of these *in silico* granulomas, *GranSim* simulates antibiotic plasma PK, tissue PK, and pharmacodynamics (PD) (71, 72). *GranSim* parameters were estimated by calibration to microbiological, immunological, plasma PK, tissue PK, and PD data from *in vitro*, rabbit, and nonhuman primate studies (see Table S1 in the supplemental material).

**(ii) Generating *in silico* granulomas.** To generate virtual granulomas for fluoroquinolone distribution prediction, we established a set of *in silico* tissue samples. We generated a collection of 100 parameter sets, capturing interindividual variation by randomly sampling host immune parameters and plasma PK parameters using Latin hypercube sampling (LHS) (73–75). LHS ensures evenly distributed, simultaneous samplings of a multidimensional parameter space. The host parameter ranges used were based on previous calibration to nonhuman primate data (71, 72). We generated two types of *in silico* tissue samples for each parameter set: cellular granuloma and caseous granuloma. We therefore treated two *in silico* tissue types for every parameter set (2 tissue types × 100 parameter sets = 200 samples) for every dose of fluoroquinolone.

**(iii) Simulating antibiotic distribution in *in silico* granulomas.** To predict fluoroquinolone distributions, we initiated 14 days of daily dosing with the dose sizes that were used in the rabbit studies (MXF 60 mg/kg and 25 mg/kg; GTX 40 mg/kg; LVX 100 mg/kg). For each of the drug/dose combinations, we simulated treatment in the 200 cellular and caseous *in silico* tissue samples. Model outputs included antibiotic concentrations in plasma, cellular granulomas, caseous granulomas, and caseum (within caseous granulomas) over time, as well as the area under the curve (AUC) for each of these tissue sections. Drug concentrations in tissue were calculated as the average total drug concentration throughout the tissue region of interest.

**(iv) Simulating antibiotic distribution in caseum.** Dynamics and AUC were estimated for caseum using a multicompartment PK model similarly to previous approaches (36). The model tracks fluoroquinolone concentrations in plasma, peripheral tissues, normal lung, cellular areas of granulomas, and caseous areas of granulomas. Fluoroquinolone concentrations in cellular and caseous areas of necrotic granulomas were measured by laser capture microdissection coupled to mass spectrometry. Fluoroquinolones are transported directly between the plasma compartment and all other compartments (see Fig. S3A in the supplemental material). We calibrated the model to laser capture microdissection measurements of fluoroquinolone concentrations in each tissue compartment using nonlinear least-squares optimization and the plasma PK parameters described above. AUC predictions were calculated by simulating 14 days of daily dosing with the dose sizes used in the rabbit efficacy studies (MXF 60 mg/kg and 25 mg/kg; GTX 40 mg/kg; LVX 100 mg/kg) and sampling 100 plasma PK parameter sets (see Fig. S3B; see also Table S3).

### Quantification of chromosome equivalents (CEQ).

CEQ analysis was conducted as described previously (33). Briefly, tissue homogenates were digested and inactivated at 80°C, and DNA was extracted using a Qiagen QIAamp 96 DNA kit (76). CEQ were quantified using a previously described protocol (77) with *sigF* primer-probe combinations (Integrated DNA Technologies) adapted from Lin et al. (49). Real-time quantitative PCR (qRT-PCR) reactions were performed, and CEQ quantification was achieved by building standard curves using serial dilutions of whole M. tuberculosis genomes prepared from broth culture (33).

### Statistical analysis.

The efficacy data presented represent a total of 37 rabbits and 896 lesions. To detect statistically significant differences in CFU, CEQ, lesion weight, or CEQ/CFU per lesion (Fig. 4A, C, and D), groups were compared using a two-tailed Mann-Whitney U (nonparametric) test, which is adequate for analyzing non-normally distributed data sets (in particular, for analyzing “zero-inflated” data sets in the case of CFU) (GraphPad Prism 7; GraphPad Software, La Jolla, CA). Proportions shown in Fig. 4B were analyzed using Fisher's exact test (two-sided for comparisons of treated versus untreated group pairs). In the CEQ/CFU ratio analyses whose results are shown in Fig. 4D, values representing lesions that harbored undetectable CEQ (the majority of which had no detectable CFU) were excluded rather than being represented by a value of zero since the value corresponding to the limit of CEQ detection was around 100 (Fig. S2). *P* values of less than 0.05 were considered statistically significant (*, *P* < 0 0.05; **, *P* < 0.01; ***, *P* < 0.001; ****, *P* < 0.0001). All data are presented as median ± 95% confidence interval, except for *in vitro* potency data and PK/PD parameters, which are shown as means and standard deviations in Fig. 2.

## Supplementary Material

Supplemental file 1

Supplemental file 2

Supplemental file 3

Supplemental file 4
