# Supplementary material for: Fluoroquinolone Efficacy against Tuberculosis Is Driven by Penetration into Lesions and Activity against Resident Bacterial Populations
Source: Antimicrob Agents Chemother. 2019 Apr 25;63(5):e02516-18. doi: 10.1128/AAC.02516-18 (PMC6496041; doi:10.1128/AAC.02516-18)
Supplement: Supplemental file 1 [file AAC.02516-18-s0001.pdf]

# Fluoroquinolone efficacy against tuberculosis is driven by penetration into lesions and activity against resident bacterial populations

Jansy Sarathy [(1) ‡], Landry Blanc [(1)‡], Nadine Alvarez-Cabrera [(1)], Paul O'Brien [(1)], Isabela Dias-Freedman [(1)], Marizel Mina [(1)], Matthew Zimmerman [(1)], Firat Kaya [(1)], Hsin-Pin Ho Liang [(1)], Brendan Prideaux [(1)], Jillian Dietzold [(2)], Padmini Salgame [(2)], Rada Savic [(3)], Jennifer Linderman [(4)], Denise Kirschner [(5)], Elsje Pienaar [(4,5) #] and Véronique Dartois [(1,2)]

[(1)] Public Health Research Institute, New Jersey Medical School, Rutgers, The State University of New Jersey, Newark, NJ, USA

[(2)] Department of Medicine, New Jersey Medical School, Rutgers, The State University of New Jersey, Newark, NJ, USA

[(3)] Department of Bioengineering and Therapeutic Sciences, University of California San Francisco, San Francisco, CA, USA

[(4)] Department of Chemical Engineering, University of Michigan, Ann Arbor, MI,

[(5)] Department of Microbiology and Immunology, University of Michigan Medical School, Ann Arbor, MI

[‡] these authors contributed equally to the present work

[#] Present Address: Purdue University, Weldon School of Biomedical Engineering, West Lafayette, IN 47907

Correspondence to: veronique.dartois@rutgers.edu

**RUNNING TITLE:** Moxifloxacin: fluoroquinolone of choice for TB?

**LIST OF SUPPLEMENTAL MATERIAL**

**Supplemental Figure S1**: Plasma pharmacokinetics of the fluoroquinolones in rabbits.

**Supplemental Figure S2**: Effect of fluoroquinolone treatment on the cumulative bacterial burden (CEQ) in uninvolved lung, cellular and necrotic lesions

**Supplemental Figure S3**: Modeling of fluoroquinolone distribution from plasma to uninvolved lung, cellular lesions and caseum.

**Supplemental Table 1.** Computational model PK and PD parameters

**Supplemental Table 2.** Protein binding of the fluoroquinolones in rabbit and human plasma

**Supplemental Table 3.** Simulation-predicted area under the curve (AUC) of the fluoroquinolones in plasma and lung lesions

**Supplemental Table 4.** Comparison of fluoroquinolone exposure achieved in rabbits on treatment with clinical PK parameters in TB patients

**Supplemental Table 5.** Number of lesions collected from each rabbit after 2 months of daily treatment

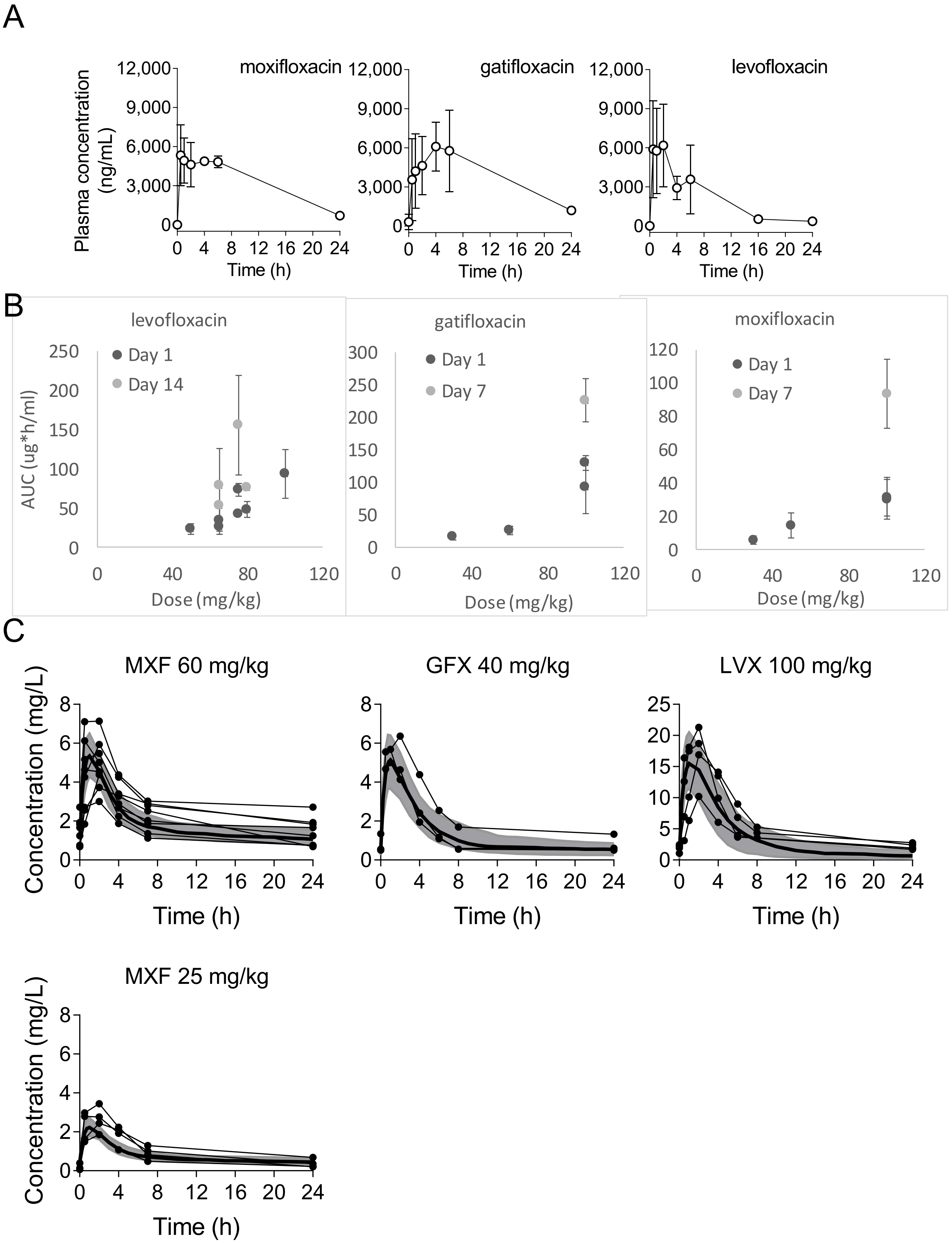
A
Plasma concentration (ng/mL)
moxifloxacin
gatifloxacin
levofloxacin
12,000
9,000
6,000
3,000
0
0 4 8 12 16 20 24
Time (h)
B
levofloxacin
Day 1
Day 14
AUC (ug*h/ml)
Dose (mg/kg)
gatifloxacin
Day 1
Day 7
moxifloxacin
Day 1
Day 7
C
MXF 60 mg/kg
GFX 40 mg/kg
LVX 100 mg/kg
MXF 25 mg/kg
Concentration (mg/L)
Time (h)

**Supplementary Figure S1:** Plasma pharmacokinetics of the fluoroquinolones in rabbits. (A) Concentration-time profile in rabbits following a single dose of 100 mg/kg MXF, 100 mg/kg GTX and 75 mg/kg LVX (n = 4, mean and standard deviations are shown). (B) Relationship between dose and plasma AUC (area under the concentration time curve) following a single oral dose and at steady state for the three fluoroquinolones. These data were used to identify the human equivalent dose administered in subsequent efficacy studies. (C) Simulated plasma concentration time profiles at the projected human-equivalent doses of MXF 400 mg, GTX 400 mg and LVX 1,000 mg. Each thin black line represents the plasma PK profile of a single rabbit. Thick black lines represent average PK profiles and shaded areas show the 95% confidence interval.

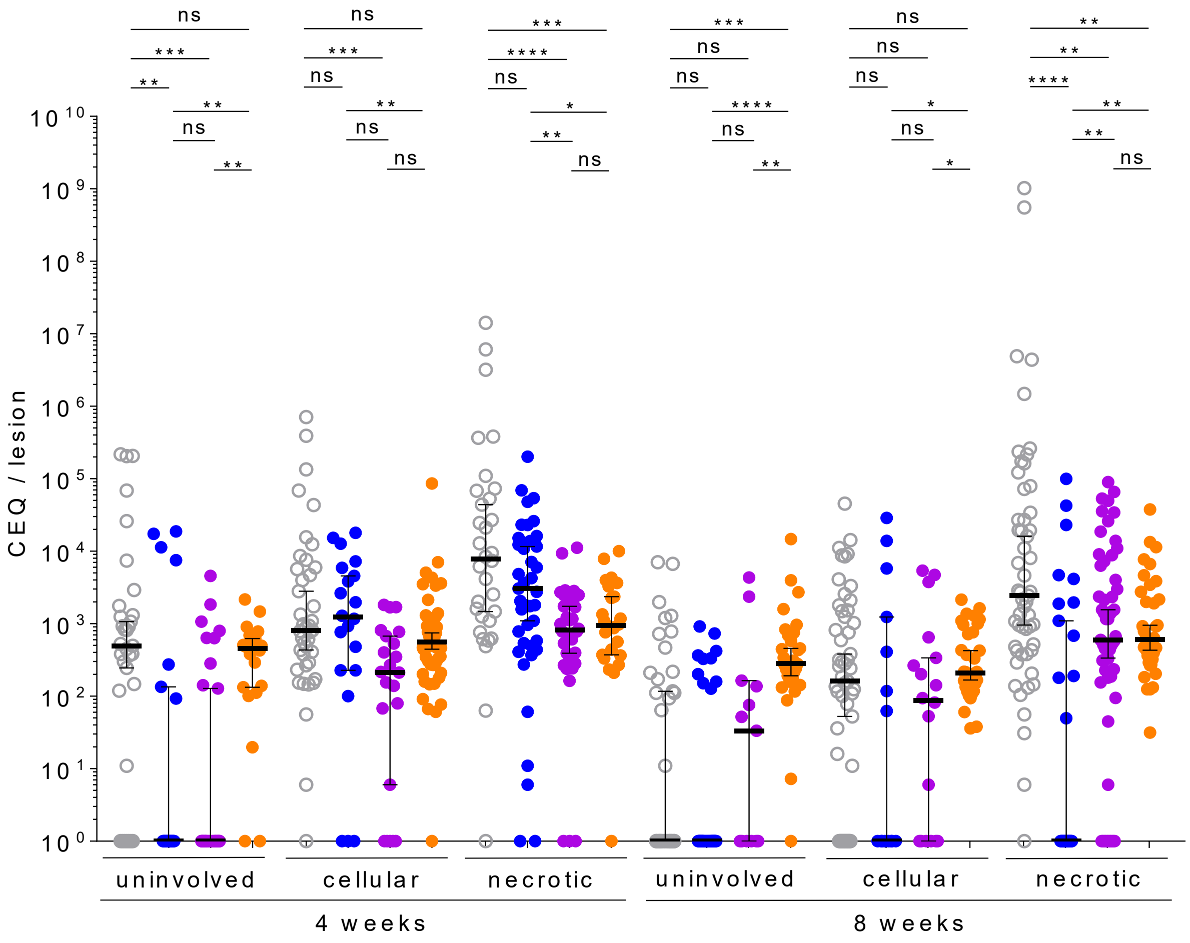


**Supplemental Figure S2.** Effect of fluoroquinolone treatment on the cumulative bacterial burden (CEQ) in uninvolved lung, cellular and necrotic lesions after 1 and 2 months of daily therapy with MXF, GTX or LVX as described. Data are presented as median ± 95% confidence interval; n = 4 to 6 rabbits or 13 to 55 lesions per treatment group, two-tailed Mann-Whitney U (non-parametric) test. *p < 0 .05, **p < 0.01, ***p < 0.001, **** p < 0.0001.

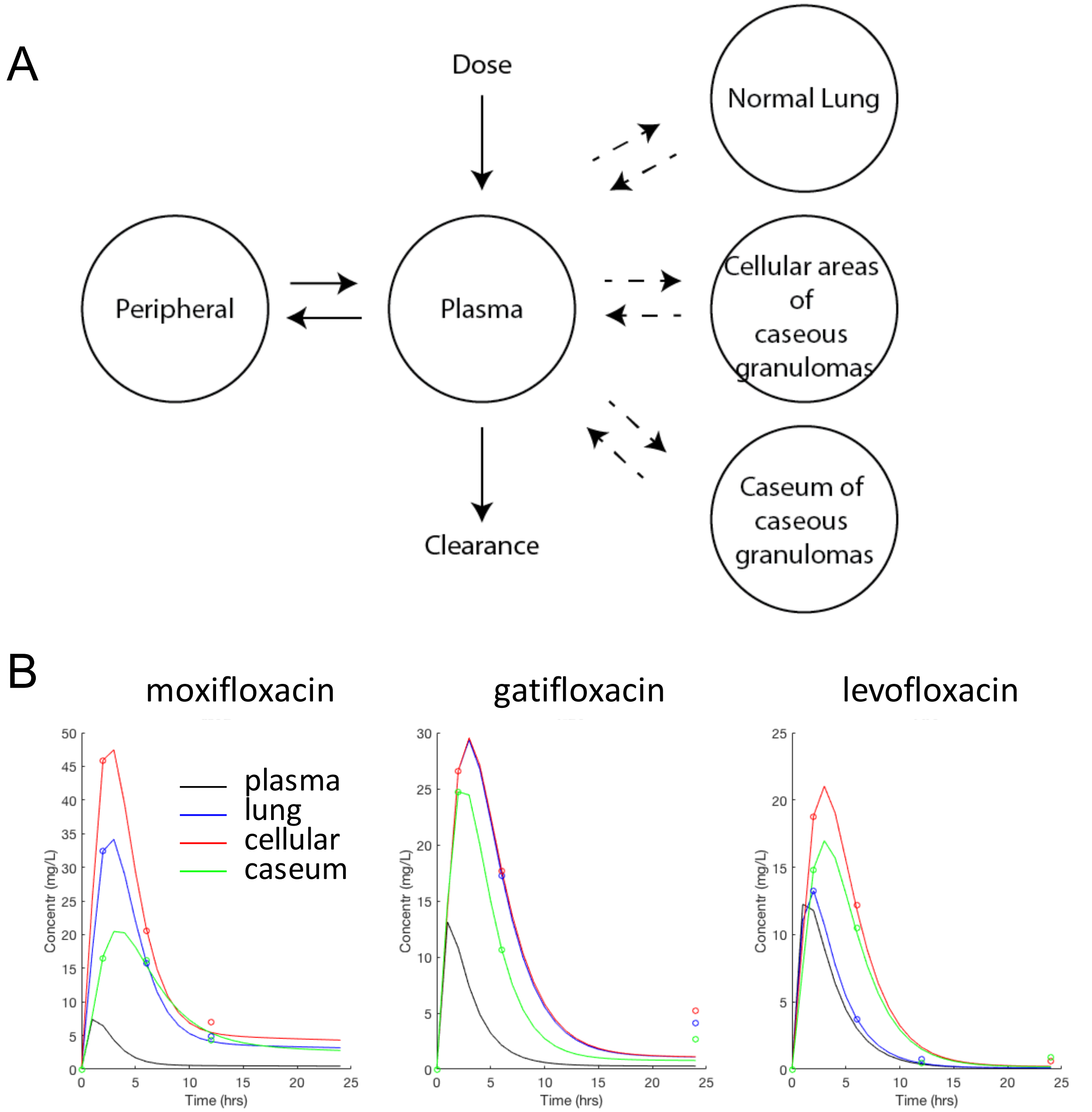


**Supplemental Figure S3**. Computational modeling of fluoroquinolone distributions from plasma to uninvolved lung, cellular lesions and caseum. (A) Multi-compartment PK model structure: fluoroquinolones are transported directly between the plasma compartment and all other compartments. Dashed arrows represent transport to small physiological compartments that do not significantly affect plasma concentrations. (B) Simulations of fluoroquinolone distribution within *in silico* granulomas and in caseum (lines), calibrated to laser capture microdissection data from rabbit lesions (circles).

**Table S1**. Computational model PK and PD parameters (see (1) for details).

| Parameter | Units | MXF | GFX | LVX |
|---|---|---|---|---|
| **Rabbit Plasma PK parameters** (mean, %CV) | | | | |
| Absorption rate constant ($k_a$) | $h^{-1}$ | 0.9, 57 | 0.46, 77 | 1.25, 50 |
| Intercompartmental clearance rate constant ($Q$) | L/h/kg | 2.86, 12 | 1.15, 44 | 0.5, 174 |
| Plasma volume of distribution ($V_p$) | L/kg | 5.35, 9 | 1.27, 39 | 3.48, 10 |
| Peripheral volume of distribution ($V_{pe}$) | L/kg | 80.8, 0.9 | 90, 1.5 | 130, 2 |
| Plasma clearance rate constant ($CL$) | L/h/kg | 1.3, 27 | 1, 61 | 0.82, 96 |
| | | | | |
| **Lung tissue PK parameters** (2) | | | | |
| Effective diffusivity ($D$) | $cm^2/s$ | $1.4x10^{-7}$ | $1.2x10^{-7}$ | $1.3x10^{-6}$ |
| Cellular accumulation ratio (3) ($a$) | - | 7.00 | 2.75 | 3.81 |
| Vascular permeability ($p$) | cm/s | $3.0x10^{-6}$ | $1.3x10^{-6}$ | $2.0x10^{-6}$ |
| Permeability coefficient ($PC$) | - | 4.87 | 3.4 | 1.56 |
| Caseum unbound fraction ($f_u$) | - | 0.26 | 0.23 | 0.36 |
| Caseum binding rate constant ($k_{fc}$) | $cu^{-1}s^{-1}$ | 0.003 | 0.006 | 0.007 |
| Epithelium binding association constant ($K_a$) | - | 0.01 | 0.01 | 0.02 |
| Epithelium binding rate constant ($k_{fe}$) | $s^{-1}$ | 0.005 | 0.01 | 0.01 |
| Cellular exit rate constant ($k_{out}$) | $s^{-1}$ | 0.11 | 0.12 | 0.16 |
| | | | | |
| **Multi-compartment PK model lung tissue PK parameters** (baseline (95% CI)) | | | | |
| Distribution rate constant plasma to normal lung ($k_{pl\text{-}lung}$) | $h^{-1}$ | 4.4 (3.9 - 5) | 1.8 (1.2 – 2.4) | 3.6 (-6 - 13) |
| Distribution rate constant normal lung to plasma ($k_{lung\text{-}pl}$) | $h^{-1}$ | 0.6 (0.5 – 0.7) | 0.5 (0.3 - 0.7) | 3.3 (-6 - 12) |
| Distribution rate constant plasma to cellular areas of granulomas ($k_{pl\text{-}cell}$) | $h^{-1}$ | 6.5 (5.9 – 7.1) | 1.8 (1.2 – 2.4) | 1.5 (1.3 – 1.8) |
| Distribution rate constant cellular areas of granulomas to plasma ($k_{cell\text{-}pl}$) | $h^{-1}$ | 0.7 (0.6 – 0.8) | 0.5 (0.3 – 0.7) | 0.7 (0.6 – 0.8) |

| | | | | |
|---|---|---|---|---|
| Distribution rate constant plasma to caseous areas of granulomas ($k_{pl\text{-}cas}$) | $h^{-1}$ | 1.8 (1.5 – 2.1) | 2.1 (1.1 – 3.1) | 1.2 (0.9 – 1.4) |
| Distribution rate constant caseous areas of granulomas to plasma ($k_{cas\text{-}pl}$) | $h^{-1}$ | 0.3 (0.2 – 0.4) | 0.8 (0.3 – 1.2) | 0.6 (0.5 – 0.7) |

**Table S2**. Protein binding of the fluoroquinolones in rabbit and human plasma

| Drug | Hu PPB [1]<br>% free (SD) | Rb PPB [1]<br>% free (SD) |
|---|---|---|
| MXF | 71.68 (0.56) | 69.53 (0.03) |
| LVX | 62.60 (0.61) | 61.02 (3.34) |
| GTX | 76.25 (2.43) | 78.75 (0.89) |

**(1)** at 2 μg/mL, or approximately the $C_{average}$ across the dosing interval

**Table S3.** Simulation-predicted area under the curve (AUC) of the fluoroquinolones in plasma and lung lesions

| Drug and dose | MXF 60 mg/kg | MXF 25 mg/kg | GTX 40 mg/kg | LVX 100 mg/kg |
|---|---|---|---|---|
| Compartment | AUC (μg*h/mL) [mean (95% CI)] | | | |
| Plasma | 45.1 (42.8 to 47.3) | 18.0 (17.1 to 18.9) | 36.0 (30.7 to 41.4) | 113.3 (89.3 to 137.4) |
| Cellular granuloma | 609.3 (584.1 to 634.5) | 243.7 (233.6 to 253.8) | 192 (166.3 to 217.6) | 325.4 (258.5 to 392.2) |
| Caseum | 276.1 (251.2 to 301.1) | 109.1 (98.68 to 119.5) | 103.1 (83.29 to 123) | 237.7 (180.7 to 294.8) |

**Table S4.** Comparison of fluoroquinolone exposure achieved in rabbits on treatment with clinical PK parameters in TB patients

| | clinical dose (mg) | Patient population | AUC (mg*h/L) in patients at steady state; mean (IQR) | $C_{max}$ (mg/L) in patients at steady state; mean (IQR) | references | rabbit dose | weeks into treatment | rabbit AUC (mg*h/L) at steady state (mean and range) | rabbit $C_{max}$ (mg/L) at steady state (mean and range) |
|---|---|---|---|---|---|---|---|---|---|
| **MXF** | 400 QD | PTB MDR-TB | 55.5 (36.6 - 78.3) | 6.1 (4.5 - 9.0) | (4) | 60 mg/kg | 3 | 43.9(30.4 - 55.3) | 4.4 (3.0 - 6.1) |
| | 400 once | DS-TB (rifaquin) | 50.8 (31.8 - 65.3) | 3.8 (2.1 - 4.6) | (5) | | | | |
| | 400 once weekly | DS-TB (rifaquin) | 46.2 (29.5 - 60.6) | 2.9 (2.0 - 3.8) | | | | | |
| | 400 twice weekly | DS-TB (rifaquin) | 45.3 (26.8 - 65.3) | 2.8 (2.0 - 4.5) | | | | | |
| | | | | | | 25 mg/kg | 3 | 13.2 (8.6 - 17.4) | 2.6 (1.9 - 3.4) |
| **GTX** | 400 QD | DS-TB (Oflotub) | 35.4 (15.2 - 80.4) | ~ 4 (2 - 6) | (6) | 40 mg/kg | 2 | 38.5 (29.5 - 56.5) | 5.7 (5.0 - 6.4) |
| | 400 QD | PTB MDR-TB | 43.4 (30.0 - 53.7) | 4.6 (3.8 - 6.4) | (4) | | | | |
| | 400 QD | TBM | 41.2 (34.2 - 85.7) | not shown | (7) | | | | |
| **LVX** | 1000 QD | PTB MDR-TB | 98.8 (84.4 - 159.6) | 10.1 (8.4 - 16.2) | (8) | 100 mg/kg | 2 | 129 (90 - 152) | 16.8 (10.2 - 21.3) |
| | 1000 QD | PTB MDR-TB | 129.1 (103.4 - 358.3) | 15.6 (8.6 - 43.0) | (9) | | | | |
| | 500 BID | TBM | 155 (81 - 284) | not shown | (7) | | | | |
| | 1000 SD | PTB MDR-TB | 96.5 (77.3 - 295.9) | 15.6 (8.6 - 42.9) | (4) | | | | |

Total AUC and $C_{max}$ values are shown since plasma protein binding is similar in rabbits and humans (**Table S2**). AUC: area under the concentration time curve, $C_{max}$: peak plasma concentration; IQR: interquartile range; PTB: pulmonary TB; DS: drug susceptible; MDR: multidrug resistant; TBM: TB meningitis; QD: once a day; BID: twice a day.

**Table S5**. Number of lesions collected from each rabbit after 2 months of daily treatment.

| | Treatment group | | | |
|---|---|---|---|---|
| FQ | MXF | GTX | LVX | Vehicle |
| Dose (mg/kg) | 60 | 40 | 100 | - |
| Rabbit 1 | 10 | 24 | 24 | 24 |
| Rabbit 2 | 6 | 9 | 17 | 21 |
| Rabbit 3 | 17 | 23 | 18 | 21 |
| Rabbit 4 | 18 | 18 | 6 | 21 |
| Rabbit 5 | | | 18 | 16 |
| Rabbit 6 | | | | 15 |

Cells highlighted in red indicate animals for which a limited number of lesions were detected and collected

## REFERENCES


1. Pienaar E, Sarathy JP, Prideaux B, Dietzold J, Dartois V, Kirschner DE, Linderman JJ. 2017. Comparing efficacies of moxifloxacin levofloxacin and gatifloxacin in tuberculosis granulomas using a multi-scale systems pharmacology approach. PLoS Comp Biol in press.
2. Ray JC, Flynn JL, Kirschner DE. 2009. Synergy between individual TNF-dependent functions determines granuloma performance for controlling Mycobacterium tuberculosis infection. J Immunol 182:3706-17.
3. Cilfone NA, Perry CR, Kirschner DE, Linderman JJ. 2013. Multi-scale modeling predicts a balance of tumor necrosis factor-alpha and interleukin-10 controls the granuloma environment during Mycobacterium tuberculosis infection. PLoS One 8:e68680.
4. Peloquin CA, Hadad DJ, Molino LP, Palaci M, Boom WH, Dietze R, Johnson JL. 2008. Population pharmacokinetics of levofloxacin, gatifloxacin, and moxifloxacin in adults with pulmonary tuberculosis. Antimicrob Agents Chemother 52:852-7.
5. Zvada SP, Denti P, Geldenhuys H, Meredith S, van As D, Hatherill M, Hanekom W, Wiesner L, Simonsson US, Jindani A, Harrison T, McIlleron HM. 2012. Moxifloxacin population pharmacokinetics in patients with pulmonary tuberculosis and the effect of intermittent high-dose rifapentine. Antimicrob Agents Chemother 56:4471-3.
6. Smythe W, Merle CS, Rustomjee R, Gninafon M, Lo MB, Bah-Sow O, Olliaro PL, Lienhardt C, Horton J, Smith P, McIlleron H, Simonsson US. 2013. Evaluation of initial and steady-state gatifloxacin pharmacokinetics and dose in pulmonary tuberculosis patients by using monte carlo simulations. Antimicrob Agents Chemother 57:4164-71.
7. Thwaites GE, Bhavnani SM, Thi Hong Chau T, Hammel JP, Torok ME, Van Wart SA, Phuong Mai P, Reynolds DK, Caws M, Thi Dung N, Tinh Hien T, Kulawy R, Farrar J, Ambrose PG. 2011. A randomised pharmacokinetic and pharmacodynamic comparison of fluoroquinolones for tuberculous meningitis. Antimicrob Agents Chemother.
8. Van't Boveneind-Vrubleuskaya N, Seuruk T, van Hateren K, van der Laan T, Kosterink JGW, van der Werf TS, van Soolingen D, van den Hof S, Skrahina A, Alffenaar JC. 2017. Pharmacokinetics of Levofloxacin in Multidrug- and Extensively Drug-Resistant Tuberculosis Patients. Antimicrob Agents Chemother 61.
9. Ghimire S, Van't Boveneind-Vrubleuskaya N, Akkerman OW, de Lange WC, van Soolingen D, Kosterink JG, van der Werf TS, Wilffert B, Touw DJ, Alffenaar JW. 2016. Pharmacokinetic/pharmacodynamic-based optimization of levofloxacin administration in the treatment of MDR-TB. J Antimicrob Chemother 71:2691-703.
